# Tranexamic acid reduces perioperative blood transfusions following open radical cystectomy – a propensity-score matched analysis

**DOI:** 10.1007/s00345-024-05168-x

**Published:** 2024-08-08

**Authors:** Luisa Egen, Karoline Keller, Hanna Saskia Menold, Allison Quan, Carl-Erik Dempfle, Jochen Johannes Schoettler, Frederik Wessels, Benjamin Meister, Thomas Stefan Worst, Niklas Westhoff, Maximilian Christian Kriegmair, Patrick Honeck, Maurice Stephan Michel, Karl-Friedrich Kowalewski

**Affiliations:** 1 Department of Urology and Urosurgery, Medical Faculty, University Medical Center Mannheim, Mannheim at Heidelberg University, Theodor-Kutzer-Ufer 1-3, 68167 Mannheim, Germany; 2 German Cancer Research Center (DKFZ) Heidelberg, Division of Intelligent Systems and Robotics in Urology (ISRU), Heidelberg, Germany; 3 DKFZ Hector Cancer Institute at the University Medical Center Mannheim, Mannheim, Germany; 4 Faculty of Health Sciences, Queen’s University, Kingston, ON Canada; 5 Coagulation Center Mannheim, Mannheim, Germany; 6 Department of Anesthesiology, Medical Faculty, University Medical Center Mannheim, Mannheim at Heidelberg University, Mannheim, Germany; 7 Urological Clinic Munich-Planegg, Germeringer Str. 32, 82152 Planegg, Germany

**Keywords:** Blood transfusion, Cystectomy, Venous thromboembolism, Tranexamic acid

## Abstract

**Purpose:**

Radical cystectomy is associated with bleeding and high transfusion rates, presenting challenges in patient management. This study investigated the prophylactic use of tranexamic acid during radical cystectomy.

**Methods:**

All consecutive patients treated with radical cystectomy at a tertiary care university center were included from a prospectively maintained database. After an institutional change in the cystectomy protocol patients received 1 g of intravenous bolus of tranexamic acid as prophylaxis. To prevent bias, propensity score matching was applied, accounting for differences in preoperative hemoglobin, neoadjuvant chemotherapy, tumor stage, and surgeon experience. Key outcomes included transfusion rates, complications, and occurrence of venous thromboembolism.

**Results:**

In total, 420 patients were included in the analysis, of whom 35 received tranexamic acid. After propensity score matching, 32 patients and 32 controls were matched with regard to clinicopathologic characteristics. Tranexamic acid significantly reduced the number of patients who received transfusions compared to controls (19% [95%-Confidence interval = 8.3; 37.1] vs. 47% [29.8; 64.8]; *p* = 0.033). Intraoperative and postoperative transfusion rates were lower with tranexamic acid, though not statistically significant (6% [1.5; 23.2] vs. 19% [8.3; 37.1], and 16% [6.3; 33.7] vs. 38% [21.9; 56.1]; *p* = 0.257 and *p* = 0.089, respectively). The occurrence of venous thromboembolism did not differ significantly between the groups (9% [2.9; 26.7] vs. 3% [0.4; 20.9]; *p* = 0.606).

**Conclusion:**

Prophylactic tranexamic administration, using a simplified preoperative dosing regimen of 1 g as a bolus, significantly lowered the rate of blood transfusion after cystectomy. This exploratory study indicates the potential of tranexamic acid in enhancing outcomes of open radical cystectomy.

**Supplementary Information:**

The online version contains supplementary material available at 10.1007/s00345-024-05168-x.

## Introduction

Radical cystectomy (RC) is the gold standard for the treatment of muscle invasive or high-risk non-muscle-invasive urothelial carcinoma [1]. As a major surgical procedure, RC carries the risk of substantial bleeding and high transfusion rates up to 60% [2]. Perioperative blood transfusions (PBT) remain a critical intervention in the management of postoperative anemia but should be administered with caution [3]. PBT can produce infectious adverse reactions, as well as immunological effects [4]. The latter is presumed to negatively affect cancer specific mortality and cancer recurrence after RC [5]. Furthermore, excessive bleeding impairs the surgeon’s visibility during surgery, and therefore poses the risk of tissue damage, further bleeding, and longer operative times [6].

Considering these challenges, there is a need for strategies to reduce bleeding and transfusion requirements. A promising approach is the administration of tranexamic acid (TXA), an antifibrinolytic drug. By inhibiting the formation of plasmin and the degradation of fibrin, TXA prevents the breakdown of blood clots, and therefore may reduce perioperative bleeding [7].

The use of TXA is already established in several sectors such as postpartum hemorrhage, trauma, cardiac and orthopedic surgery, and coagulopathies [7]. A more recent realm of application is the prophylactic administration for major abdominal surgery. Current literature strongly suggests that the perioperative application of TXA decreases the requirement for PBT and adverse effects associated with major bleeding events [8]. Despite this compelling evidence, potential adverse effects and known contraindications should not be disregarded. The most pertinent side effect is the formation of blood clots and consecutive thrombosis or embolism. For patients with a history of blood clots or known risk factors for thrombosis, TXA administration should be evaluated with additional caution [8].

Regarding urologic surgeries, a systematic review and meta-analysis of randomized controlled trials (RCT) by Lin et al. found TXA to reduce transfusion rates and blood loss in numerous procedures, namely percutaneous nephrolithotomy, transurethral resection of bladder or prostate, and radical prostatectomy [9]. In accordance with most reviews on perioperative TXA application, there was a considerable heterogeneity in terms of dosage and application regimens [8–10]. Depending on the type of procedure and surgeon preference, the route of application is either oral, intravenous, or topical. Different dosing regimens include a single dose (ranging from 0.5 to 2 g or weight-adjusted), multiple applications, continuous infusion, or a combination of the above [10]. Notably, there is an RCT registered in 2013, investigating the effects of TXA during RC, but has yet to publish results [11]. This lack of accrual and reporting emphasizes the need for the publication of existing retrospective data.

To our knowledge this is the first study to analyze the prophylactic use of TXA with a simplified dosing regimen of 1 g preoperatively in the scenario of RC. The objective of this study is to provide evidence concerning the potential of TXA to prevent bleeding during open RC, with the ultimate goal of identifying a tool for the reduction of PBT in patients undergoing one of the most intricate urologic procedures.

## Materials and methods

### Study design

An institutional modification of the clinical standard protocol for open RC was implemented at the Department of Urology, University Medical Center Mannheim in November 2022. After ruling out contraindications (Table 1), each patient received an intravenous administration of 1 g TXA half an hour before open RC, as previously described by Devereaux et al [8]. The control group comprised of patients who underwent RC at the University Medical Center Mannheim from January 2019 to June 2023. Perioperative thrombosis prophylaxis was carried out according to ERAS standards, with heparin prophylaxis starting on the evening of surgery and continuing for a total four weeks postoperatively [12]. Patient data was retrospectively retrieved from an institutional review board approved database (2008–310 N-MA) with consideration of the predefined inclusion and exclusion criteria (see Supplementary Table 1).

### Data acquisition

Patient demographics included age, sex, body mass index (BMI), preoperative creatinine and hemoglobin, history of coronary artery disease, venous thromboembolism (VTE), neoadjuvant chemotherapy (NAC), American Association of Anesthesiologists [13] (ASA) score, and Charlson Comorbidity Score. VTA included deep vein thrombosis and pulmonary embolism, transitory ischemic attack (TIA), or stroke. Tumor stage, nodal status, positive surgical margin (PSM) status, and number of resected lymph nodes were retrieved from histopathological reports. The RCs were performed by surgeons with different levels of expertise that was scored according to the number of completed RCs (level 1: 0–10, level 2: >10–20, level 3: >20–50, level 4: >50–100, and level 5: >100 RCs). Furthermore, the type of urinary diversion and rate of continent diversions were reported. The most important outcome parameters were transfusion rates – subdivided by intraoperative, postoperative and total rates (including all patients receiving intra- and/or postoperative transfusions to account for patients receiving transfusions at both occasions) – and complication rates – including intraoperative, postoperative overall and postoperative major (Clavien Dindo classification [14] > 2) complications, Comprehensive Complication Index [15] (CCI), and occurrence of VTE. Additionally, postoperative hemoglobin (Hb) on the first postoperative day (POD1), Hb drop (preoperative minus POD1), length of stay (LOS), 30-day resubmission, and 30-day mortality were reported.

### Statistical analysis

Patient data was compiled in an Excel spreadsheet and analyzed using R, version 4.3.1 (R-Studio, R-Foundation for Statistical Computing, Vienna, Austria). Patients who received TXA and controls were balanced using propensity score matching using the nearest-neighbor method with a 1:1 ratio. Covariates included preoperative Hb, NAC, tumor stage (≤ T2 vs. > T2), and surgeon experience. Continuous variables are provided as mean ± standard deviation (SD), ordinal variables as median and interquartile range (IQR), and binary data as absolute and relative frequencies with respective 95%-confidence intervals (CI). Continuous and categorical variables were compared using the t-test or Mann-Whitney-U-test, and the chi-squared test, respectively. A p-value ≤ 0.05 was considered statistically significant.

## Results

### Baseline characteristics and clinicopathologic findings

In total, 420 patients were incorporated in the analysis, including 35 patients who received TXA. Prior to propensity score matching, the TXA and control groups differed significantly in ASA score (*p* < 0.001), administration of NAC (TXA: 31% (*n* = 11), control: 14% (*n* = 55), *p* = 0.015), tumor stage (*p* = 0.04), and surgeon experience (*p* < 0.001). After propensity score matching, the two groups were balanced with respect to clinicopathologic characteristics (Table 1).

**Table 1:** Clinicopathologic data

	Overall	Unmatched			Matched		
	(*n* = 420)	TXA (*n* = 35)	Control (*n* = 385)	*p*-value	TXA (*n* = 32)	Control (*n* = 32)	*p*-value
Female sex, n (%)	102 (24)	6 (17)	96 (25)	*0.410*	5 (16)	11 (34)	*0.149*
Age (years), mean (SD)	69 (10)	69 (10)	69 (10)	*0.815*	68 (10)	72 (9)	*0.141*
BMI (kg/m2), mean (SD)	27 (5)	28 (5)	27 (5)	*0.446*	28 (5)	27 (5)	*0.294*
Preoperative creatinine (mg/dl), mean (SD)	1.1 (0.4)	1.1 (0.4)	1.1 (0.4)	*0.506*	1.1 (0.3)	1.0 (0.4)	*0.611*
Preoperative hemoglobin (g/dl), mean (SD)	13.2 (2.0)	13.2 (2.0)	13.3 (2.0)	*0.794*	13.2 (2.1)	13.0 (2.1)	*0.764*
ASA, n (%)				***< 0.001***			*0.136*
1	29 (7)	9 (26)	20 (5)		8 (25)	2 (6)	
2	235 (56)	16 (46)	219 (57)		15 (47)	20 (63)	
3	153 (36)	9 (26)	144 (37)		8 (25)	10 (31)	
4	3 (1)	1 (3)	2 (1)		1 (3)	0 (0)	
Charlson Score, median (IQR)	5 (4;6)	5 (4;6)	5 (4;6)	*0.951*	6 (4;6)	6 (4;6)	*0.180*
Coronary artery disease, n (%)	59 (14)	6 (17)	53 (14)	*0.767*	5 (16)	5 (16)	*1.000*
History of DVT/ PE, n (%)	11 (3)	0 (0.0)	11 (3)	*0.645*	0 (0.0)	2 (6)	*0.472*
History of TIA/ stroke, n (%)	32 (8)	3 (9)	29 (8)	*1.000*	3 (9)	4 (13)	*1.000*
NAC, n (%)	66 (16)	11 (31)	55 (14)	***0.015***	11 (34)	12 (38)	*1.000*
Tumor stage, n (%)				***0.004***			*0.141*
pT0	47 (11)	12 (34)	35 (9)		11 (34)	5 (16)	
pTis	6 (1)	0 (0)	6 (2)		0 (0)	2 (6)	
pT1	65 (16)	4 (11)	61 (16)		3 (9)	5 (16)	
pT2	80 (19)	7 (20)	73 (9)		7 (22)	3 (9)	
pT3	103 (25)	6 (17)	97 (25)		5 (16)	11 (34)	
pT4	119 (28)	6 (17)	113 (29)		6 (19)	6 (19)	
Nodal status, n (%)				*0.275*			*0.443*
pN0	320 (76)	22 (63)	297 (77)		20 (63)	24 (75)	
pN1	36 (9)	6 (17)	30 (8)		5 (16)	3 (9)	
pN2	62 (15)	7 (20.0)	55 (13)		7 (22)	4 (13)	
pN3	2 (1)	0 (0)	2 (1)		0 (0)	1 (3)	
PSM, n (%)	32 (8)	5 (14)	27 (7)	*0.222*	5 (16)	2 (6)	*0.423*
Urinary diversion, n (%)				*0.720*			*0.551*
Conduit	217 (52)	21 (60)	196 (51)		18 (56)	20 (63)	
Neobladder	162 (39)	12 (34)	150 (39)		12 (38)	12 (38)	
Pouch	20 (5)	1 (3)	19 (5)		1 (3)	0 (0)	
Ureterocutaneostomy	21 (5)	1 (3)	20 (5)		1 (3)	0 (0)	
Continent diversion, n (%)	182 (43)	13 (37)	169 (44)	*0.553*	13 (41)	12 (38)	*1.000*
Operative time (min), mean (SD)	213 (75)	228 (99)	212 (73)	*0.240*	224 (99)	199 (76)	*0.271*
Surgeon’s experience, n (%)				***< 0.001***			*1.000*
0–10	20 (5)	5 (14)	15 (4)		5 (16)	5 (16)	
> 10–20	5 (1)	4 (11)	1 (1)		1 (3)	1 (3)	
> 20–50	29 (7)	0 (0)	29 (7)		0 (0)	0 (0)	
> 50–100	76 (18)	1 (3)	75 (20)		1 (3)	1 (3)	
> 100	290 (69)	25 (71)	265 (69)		25 (78)	25 (78)	

SD - standard deviation, ASA - American Association of Anesthesiologists score, IQR - interquartile range, DVT - deep vein thrombosis, PE - pulmonary embolism, TIA - transitory ischemic attack, NAC - neoadjuvant chemotherapy, PSM - positive surgical margin

### Postoperative outcomes

**Fig. 1:**
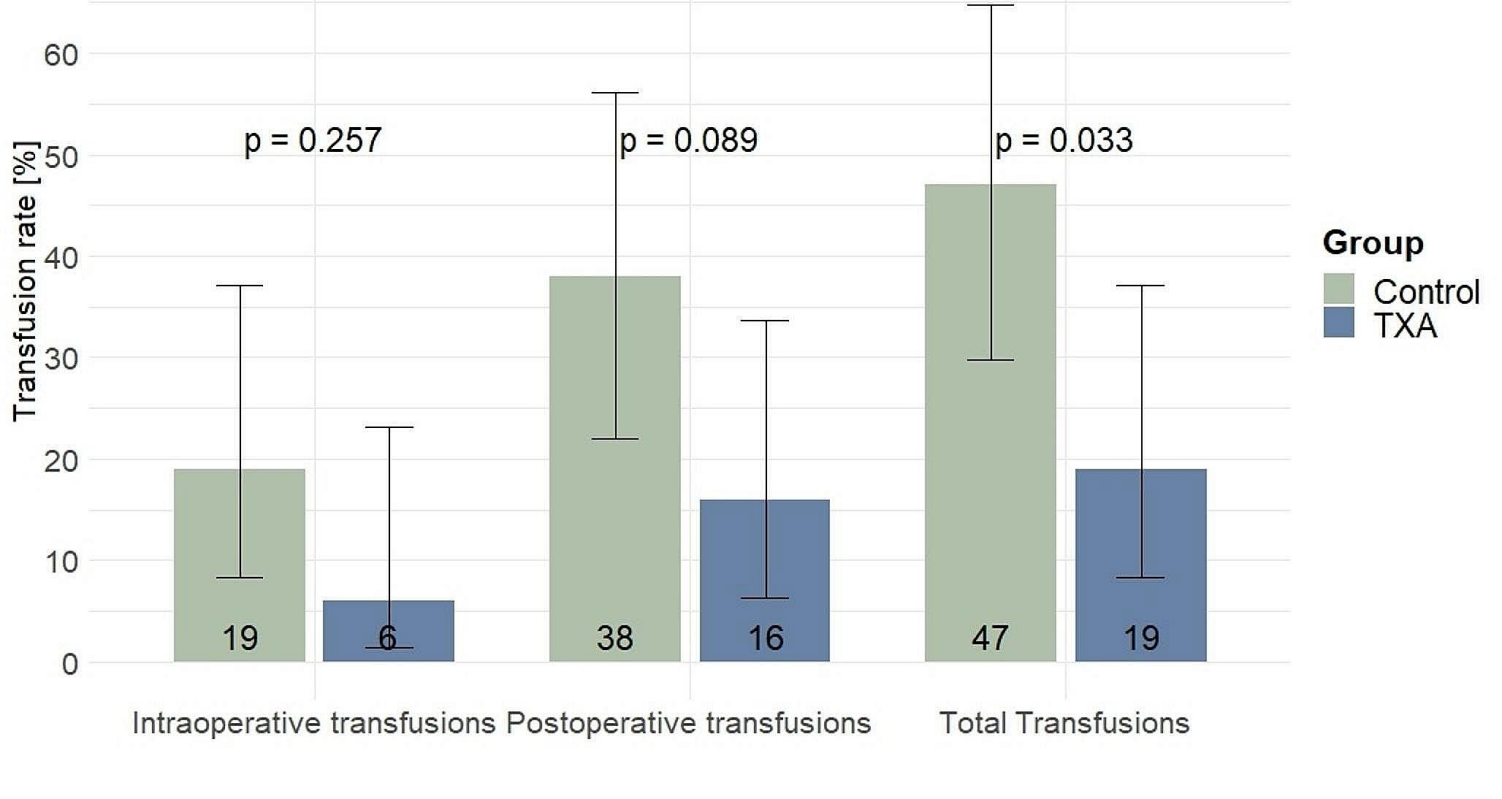
Grouped bar plots and 95%-Confidence Intervals of perioperative transfusion rates of the matched cohort, stratified by Tranexamic Acid (TXA) and control group. A p-value < 0.05 was considered statistically significant

The proportion of patients who received PBT was significantly smaller in the TXA group (TXA: 19% [95%-CI = 8.3; 37.1] vs. control: 47% [29.8; 64.8], *p* = 0.033). Intraoperative and postoperative transfusion rates were lower in the TXA than in the control group, though not on a statistically significant level (TXA: 6% [1.5; 23.2] vs. control: 19% [8.3; 37.1], *p* = 0.257, and 16% [6.3; 33.7] vs. 38% [21.9; 56.1], *p* = 0.089, respectively). No side effects of TXA administration were recorded. Perioperative complications, especially with regard to VTE did not differ between both groups (TXA: 9% [2.9; 26.7] vs. control: 3% [0.4; 20.9], *p* = 0.606). LOS, 30-day resubmission, and 30-day mortality rates were similar between TXA patients and controls (See Table 2 and Fig. 1).

**Table 2:** Perioperative outcomes

	Overall	Unmatched			Matched			
	(*n* = 420)	TXA (*n* = 35)	Control (*n* = 385)	*p*-value	TXA (*n* = 32)	Control (*n* = 32)	*p*-value	
**Transfusions, n (%)**								
Intraoperative	43 (10)	3 (9)	40 (10)	*0.961*	2 (6)	6 (19)	*0.257*	
Postoperative	98 (23)	7 (20)	91 (24)	*0.781*	5 (16)	12 (38)	*0.089*	
Total (intra- and/or postoperative	117 (28)	9 (26)	108 (28)	*0.922*	6 (19)	15 (47)	***0.033***	
**No. of Transfusions per patient, mean (SD)**								
Intraoperative	1.7 (1.0)	1.7 (1.2)	1.7 (1.0)	*0.899*	2.0 (1.4)	1.5 (0.6)	*0.453*	
Postoperative	2.1 (1.7)	2.1 (1.4)	2.1 (1.4)	*0.936*	2.0 (1.2)	1.5 (0.5)	*0.244*	
Hb POD1 (g/dl), mean (SD)	9.5 (1.6)	9.9 (1.7)	9.4 (1.6)	*0.194*	9.9 (1.6)	9.8 (1.6)	*0.662*	
Hb drop (g/dl), mean (SD)	3.8 (1.8)	3.4 (1.4)	3.8 (1.8)	*0.141*	3.3 (1.4)	3.3 (1.7)	*0.968*	
**Complications**								
Intraoperative, n (%)	21 (5)	1 (3)	20 (5)	*0.840*	0 (0)	1 (3)	*1.000*	
Postoperative, n (%)	307 (73)	24 (69)	283 (74)	*0.666*	21 (66)	27 (84)	*0.149*	
Major, n (%)	118 (28)	12 (34)	106 (28)	*0.513*	11 (34)	8 (25)	*0.584*	
CCI, mean (SD)	25 (21)	23 (20)	24 (21)	*0.767*	22 (21)	26 (19)	*0.468*	
VTE, n (%)	14 (3)	4 (11)	10 (3)	***0.022***	3 (9)	1 (3)	*0.606*	
LOS (days), mean (SD)	18 (10)	18 (9)	18 (10)	*0.963*	18 (9)	18 (8)	*0.837*	
30-Day Resubmission	74 (18)	6 (17)	68 (18)	*0.828*	5 (16)	5 (16)	*1.000*	
30-Day Mortality	6 (1)	0 (0)	6 (2)	*1.000*	0 (0)	0 (0)	*NA*	

Hb - hemoglobin, POD1 - postoperative day 1, SD - standard deviation, CCI - Comprehensive Complications Index, VTE – venous thromboembolism, LOS - length of stay

## Discussion

In this retrospective trial, TXA was identified to significantly reduce total transfusion rates for patients undergoing open RC compared to historic controls. When considered separately, intraoperative and postoperative transfusions were lower in the TXA group, although not at a statistically significant level. There was no significant difference in the occurrence of VTE between both groups.

In theory, TXA carries three substantial benefits in prophylactic, perioperative use: (1) faster recovery due to less blood loss; (2) improved oncological outcomes by avoiding potential immunological effects of transfusions [4]; and (3) reduced economic burden due to the lower costs of TXA compared to red blood cell concentrates. The application of TXA for the reduction of intraoperative bleeding and subsequent need for PBT has been subject to various medical and surgical investigations.

Large-scale obstetric trials show a lower risk of postpartum hemorrhage after cesarean Sect. [16] and decreased mortality in women with postpartum hemorrhage (relative risk (RR) = 0.81; 95%-CI = 0.65-1.00) [17]. Furthermore, TXA is widely recognized in trauma care [18] and cardiac surgery, resulting in a decreased risk of death (RR = 0.91; 95%-CI = 0.85–0.97) and less bleeding requiring reintervention [19]. Devereaux et al. performed an RCT comparing the application of TXA to placebo in non-cardiac surgery. In the TXA group, the RR for various bleeding outcomes, including bleeding associated with death, major bleeding, and PBT, was consistently lowered by 25% [8].

Furthermore, there are two recent systematic reviews and meta-analyses concerning urological surgeries [10] and urologic RCTs [9]. The latter evaluated seven studies on percutaneous nephrolithotomy, five on prostatectomy, and one on transurethral resection of the prostate/bladder, indicating transfusion rates of 8.4% (66/783) and 16.3% (129/793) in the TXA and placebo groups, respectively (odds ratio = 0.43, 95%-CI = 0.29–0.63) [9]. Kim et al. investigated eleven trials on percutaneous nephrolithotomy, ten on transurethral resection of the prostate, three on prostatectomy and one on RC. Again, TXA administration led to a reduced necessity for PBT (RR = 0.46, 95%-CI = 0.36–0.59, *p* = 0.3) [10]. There is only one other study analyzing the prophylactic application of TXA during open RC [20]. In this retrospective study, Zaid et al. included 103 patients who had received TXA as a bolus (10 mg/kg) followed by continuous infusion (2 mg/kg/hour) during surgery and performed a matched analysis. The rates of PBT were at 31.1% and 57.5% (*p* = 0.0001) in the TXA group and the matched control group, respectively. Timing of PBT – intra- or postoperatively only, or both – did not differ between the groups (*p* = 0.19) [20]. Findings from Zaid et al. align with this study’s results. Similarly, lower transfusion rates in the TXA group were found when examining intraoperative or postoperative transfusions individually, though these were not statistically significant. However, the number of patients in the cohort may have been too small to detect significant differences. Nevertheless, the analysis of all the of patients who received transfusion presented a significant reduction in PBT regardless of the timing of administration. At 19% and 47% in the TXA and control groups, respectively, the percentage of PBT was lower than reported in the study by Zaid et al. In a recently published conference abstract, Ahmed et al. reported on a study that included 2,862 patients who underwent cystectomy, of which 479 patients received perioperative TXA. They concluded that TXA-recipients experienced significantly lower intraoperative blood loss (*p* = 0.03) and a reduced rate of PBT (31% vs. 49%, *p* < 0.01) [21]. The exact dosing regimen is not given in the abstract, therefore the publication of the full study must be awaited. In a large-scale surgical meta-analysis by Ker et al. with more than 100 RCTs and over 10,000 patients included, it was consistently shown that the perioperative use of TXA decreased the likelihood of transfusion by 38% [22].

While the effectiveness of TXA is widely acknowledged, it is essential to note that dosing and routes of administration vary considerably. Kim et al. reported that the primary method of administering TXA involved an intravenous dose of at least 1 g, as observed in 14 of the 26 included studies. In order to mitigate potential impacts of differing administration methods on the results, a sensitivity analysis was conducted by excluding papers deviating from this regimen in the meta-analysis. Yet, the correlation between TXA administration and reduced estimated blood loss (mean difference − 131.5 ml, 95%-CI: -211.6 to -51.40, *p* < 0.001), Hb decrease (mean difference − 0.52 g/dl, 95%-CI: -0.67 to -0.37, *p* < 0.09), and decreased transfusion risk (RR = 0.34, 95%-CI: 0.21–0.56, *p* < 0.35) remained consistent [10]. We deliberately applied a simple dosing regimen of 1 g administered preoperatively as a bolus to simplify the applicability and achieve low-threshold implementation into daily practice.

Regarding TXA administration, it is imperative to address potential adverse events, notably thromboembolic events and seizures, which, although rare, are well-documented. A recent extensive meta-analysis, encompassing a total of 234 studies with 102,681 patients, found no evidence indicating that TXA increases the risk of VTE (RR = 1.04, 0.92–1.17), seizures (RR = 1.18, 0.91–1.53), acute coronary syndrome (RR = 0.88, 0.78-1.00), or stroke (RR = 1.12, 0.98–1.27). For seizures, a dosage-related effect was observed in association with TXA application (*p* = 0.01) [23]. Accordingly, another meta-analysis by the same authors focusing on the perioperative use of TXA found no impact of TXA on postoperative thrombotic events, including myocardial infarction, stroke, deep vein thrombosis, or pulmonary embolism [22]. The study by Deveraux et al. aligns with these findings, demonstrating comparable VTE rates after TXA and placebo administration [8]. However, the study failed to establish noninferiority of TXA concerning VTE, emphasizing the need for more careful considerations [8]. From a urological standpoint, Kim et al. found ten studies that reported on thromboembolic events. Seven studies showed no thrombotic adverse events in either group, while a meta-analysis of three studies reporting > 0 thrombotic adverse events found no significant difference in VTE risk between the TXA group and patients receiving placebo (RR 0.86, 95% CI 0.31–2.41, *p* = 0.31, I2 = 14%) [10]. However, caution should be exercised with RC patients. A study by Hammond et al. found that RC patients have the second highest risk of postoperative VTE of all cancer types studied, making careful evaluation crucial [24]. Nonetheless, the retrospective study by Zaid et al. on 103 RC patients, along with our findings, did not find a higher 30-day VTE risk associated with TXA [20]. Regardless of TXA application, as stated by Chiang et al. standardized implementation of VTE prophylaxis during in-patient and post discharge period are indispensable [12].

This study is not devoid of limitations. The TXA cohort consists of a rather small sample size that may limit the generalizability of the results. This is a consequence of the size of the initial data due to a noteworthy institutional change. While the large effect of TXA administration is remarkable and warrants further investigation, this study must be considered as hypothesis-generating. We recognize that the reported ‘Hb drop’ was not adjusted for intraoperative transfusions. While the intraoperative transfusions are low in both groups, this variable should still be regarded with caution. Furthermore, this study focused on open RC, given that at the University Medical Center Mannheim and in Germany in general, RC is still predominantly performed using the open approach. Therefore, the results may not be directly applicable to robotic RC. The retrospective nature of the study also introduces inherent limitations. However, as existing meta-analyses on TXA application in urologic surgeries prove, RC is highly underrepresented and high-level evidence is lacking. There is only one other retrospective analysis focusing on RC from 2016, preceding this study. Therefore, this study contributes to the limited body of knowledge thus far. Remarkably, the TACT-trial [11], an RCT evaluating TXA versus placebo to prevent PBT during RC was registered and initiated in 2013. However, results are still pending. To fill this gap, this analysis serves as preparation for an upcoming RCT at the University Medical Center Mannheim that investigates effect estimates for sample size calculation.

## Conclusions

In summary, TXA potentially reduces blood transfusions in patients undergoing open RC. Available literature already indicates the benefits for a widespread use of TXA, thus high-quality evidence for the use of TXA during RC is needed. On this basis, the inclusion of TXA in current ERAS guidelines should be targeted.

## Electronic supplementary material

Below is the link to the electronic supplementary material.

Supplementary Material 1

## Abbreviations

ASA: American association of anesthesiologists
BMI: Body mass index
CCI: Comprehensive Complication Index
CI: Confidence interval
Hb: Hemoglobin
IQR: Interquartile range
LOS: Length of stay
NAC: Neoadjuvant chemotherapy
PBT: Perioperative blood transfusion
POD: Postoperative day
PSM: Positive surgical margin
RC: Radical cystectomy
RCT: Randomized controlled trial
RR: Relative risk
SD: Standard deviation
TIA: Transitory ischemic attack
TXA: Tranexamic acid
VTE: Venous thromboembolism
