# Supplementary material for: Tranexamic acid reduces perioperative blood transfusions following open radical cystectomy – a propensity-score matched analysis
Source: World J Urol. 2024 Aug 8;42(1):477. doi: 10.1007/s00345-024-05168-x (PMC11310255; doi:10.1007/s00345-024-05168-x)
Supplement: Supplementary file 1 — Supplementary Material 1 [file 345_2024_5168_MOESM1_ESM.docx]

## SUPPLEMENTARY INFORMATION – World Journal of Urology

## Tranexamic acid reduces perioperative blood transfusions following open radical cystectomy – A propensity-score matched analysis

Luisa Egen^1,2,3^, Karoline Keller^1^, Hanna Saskia Menold^1^, Allison Quan^1,4^, Carl-Erik Dempfle^5^, Jochen Johannes Schoettler^6^, Frederik Wessels^1^, Benjamin Meister^1^, Thomas Stefan Worst^1^, Niklas Westhoff^1^, Maximilian Christian Kriegmair^7^, Patrick Honeck^1^, Maurice Stephan Michel^1^, Karl-Friedrich Kowalewski^1,2,3^

^1^Department of Urology and Urosurgery, University Medical Center Mannheim, Medical Faculty Mannheim at Heidelberg University, Germany.

^2^ German Cancer Research Center (DKFZ) Heidelberg, Division of Intelligent Systems and Robotics in Urology (ISRU), Heidelberg Germany.

^3^ DKFZ Hector Cancer Institute at the University Medical Center Mannheim, Mannheim, Germany.

^4^ Queen’s University, Faculty of Health Sciences, Kingston, Ontario, Canada

^5^ Coagulation Center Mannheim, Mannheim, Germany.

^6^ Department of Anesthesiology, University Medical Center Mannheim, Medical Faculty Mannheim at Heidelberg University, Germany

^7^ Urological Clinic Munich-Planegg, Germeringer Str. 32, 82152 Planegg, Germany

Correspondence to:

Karl-Friedrich Kowalewski, M.D.

Department of Urology and Urosurgery

University Medical Center Mannheim

Theodor-Kutzer-Ufer 1-3, 68167 Mannheim, Germany

P: +49 621 383 1588

F: +49 621 383 2076

E-Mail: Karl-Friedrich.Kowalewski@umm.de

| Inclusion criteria | Exclusion criteria |
| --- | --- |
| - Patients eligible for open RC at Mannheim University Medical Center - Oncologic indication (urothelial carcinoma) - Adult (>18 years) | - Contraindication for TXA application: - <12 months prior to surgery: deep vein thrombosis, pulmonary embolism, coronary stent insertion - Known thrombophilia (e.g. antiphospholipid antibody syndrome, activated protein C resistance (APCR), prothrombin G20210A, protein C deficiency, protein S deficiency, thrombotic thrombocytopenic purpura) - Known hypersensitivity to TXA - Therapeutic TXA application during surgery - Incomplete data |

Supplementary Table 1: Summary of inclusion and exclusion criteria (RC – radical cystectomy, TXA – tranexamic acid).
